# A Graph Contrastive Learning Method for Enhancing Genome Recovery in Complex Microbial Communities

**DOI:** 10.3390/e27090921

**Published:** 2025-08-31

**Authors:** Guo Wei, Yan Liu

**Affiliations:** 1 Department of Computer Science, Yangzhou University, Yangzhou, 225100, China; weiguo@smail.nju.edu.cn; 2 State Key Laboratory of Pharmaceutical Biotechnology, School of Life Sciences, Nanjing University, Nanjing 210023, China

**Keywords:** information integration, entropy, mutual information, canonical correlation analysis, genome binning

## Abstract

Accurate genome binning is essential for resolving microbial community structure and functional potential from metagenomic data. However, existing approaches—primarily reliant on tetranucleotide frequency (TNF) and abundance profiles—often perform sub-optimally in the face of complex community compositions, low-abundance taxa, and long-read sequencing datasets. To address these limitations, we present MBGCCA, a novel metagenomic binning framework that synergistically integrates graph neural networks (GNNs), contrastive learning, and information-theoretic regularization to enhance binning accuracy, robustness, and biological coherence. MBGCCA operates in two stages: (1) multimodal information integration, where TNF and abundance profiles are fused via a deep neural network trained using a multi-view contrastive loss, and (2) self-supervised graph representation learning, which leverages assembly graph topology to refine contig embeddings. The contrastive learning objective follows the InfoMax principle by maximizing mutual information across augmented views and modalities, encouraging the model to extract globally consistent and high-information representations. By aligning perturbed graph views while preserving topological structure, MBGCCA effectively captures both global genomic characteristics and local contig relationships. Comprehensive evaluations using both synthetic and real-world datasets—including wastewater and soil microbiomes—demonstrate that MBGCCA consistently outperforms state-of-the-art binning methods, particularly in challenging scenarios marked by sparse data and high community complexity. These results highlight the value of entropy-aware, topology-preserving learning for advancing metagenomic genome reconstruction.

## 1. Introduction

Metagenomics enables the comprehensive characterization of microbial communities by directly sequencing the collective genomic content of environmental samples, providing a cultivation-independent approach to studying microbial diversity and function [1,2,3]. This methodology has become foundational across a wide range of disciplines, including human health, agriculture, food safety, and climate science [4,5,6]. In contrast to traditional culture-dependent techniques, metagenomics circumvents the limitations imposed by microbial cultivability, allowing for the discovery and functional annotation of a vast array of previously inaccessible microorganisms and their metabolic potential [7,8].

Over the past decades, the field of computational metagenomics has witnessed significant advancements, resulting in the development of key analytical tools such as metagenomic assembly [9], contig binning [10,11], and microbial feature profiling [12]. Among these, contig binning is particularly critical; it seeks to cluster DNA fragments derived from the same or phylogenetically related genomes into distinct bins. This step is essential for reconstructing microbial community structure, elucidating metabolic potential, and identifying novel microbial lineages with potential biotechnological value [13]. Nonetheless, the high complexity, sparsity, and heterogeneity characteristics [14] of environmental metagenomic datasets continue to pose substantial challenges to achieving both accurate and scalable genome binning.

To overcome the challenges of metagenomic binning, modern algorithms increasingly incorporate diverse features such as guanine–cytosine (GC) content, k-mer frequencies (i.e., the count or normalized occurrence of all possible nucleotide subsequences of length k), and coverage profiles, often employing multimodal data fusion strategies. Binning methods are typically categorized into three groups [15]: composition-based, abundance-based, and hybrid approaches. Among these, hybrid models generally achieve better performance by leveraging complementary signals across feature types. However, the effective integration of heterogeneous features remains a core challenge, particularly in capturing non-linear dependencies and ensuring scalability. Early methods such as CONCOCT [16] relied on dimensionality reduction (e.g., principal component analysis [17]) over composition and coverage features, followed by clustering via a Gaussian Mixture Model (GMM) [18]. MaxBin2 [19] adopted a probabilistic framework based on tetranucleotide frequencies and coverage, using an Expectation-Maximization [20] algorithm to infer genome assignments. MetaBAT2 [21] computed pairwise similarities between contigs using k-mer and coverage information and employed a modified Label Propagation Algorithm for clustering. These approaches laid the groundwork but often suffered from normalization sensitivity, limited flexibility, or shallow integration strategies. More recent methods aim to improve robustness and resolution. Binny [22] uses multiple k-mer profiles and coverage data in an iterative, non-linear dimensionality reduction pipeline, followed by clustering via HDBSCAN [23]. MetaDecoder [24] employs a two-phase approach: initial clustering with a modified Dirichlet Process Gaussian Mixture Model (DPGMM) [25], refined by a semi-supervised probabilistic model based on k-mer frequencies and a modified GMM. MetaBinner [26], in contrast, takes an ensemble learning approach, combining partial seed k-means across multiple feature types and integrating results using a two-stage ensemble framework. Despite these advances, many methods still fall short in capturing complex, high-order dependencies across features, particularly in high-dimensional biological data. Moreover, few frameworks incorporate information-theoretic principles—such as entropy or mutual information—which can provide theoretical guidance for feature selection, redundancy reduction, and improved representation learning.

In recent years, deep learning has markedly advanced metagenomic binning by enabling the extraction of informative representations from complex, high-dimensional data sources [27,28]. For instance, variational autoencoders (VAEs) have been employed to jointly embed k-mer composition and abundance features, while semi-supervised frameworks have incorporated auxiliary constraints to guide representation learning. Moreover, contrastive multi-view learning strategies [29] have improved the integration of heterogeneous modalities by aligning different feature views. However, despite these innovations, most existing methods rarely incorporate information-theoretic principles, such as mutual information maximization or entropy-based regularization, to explicitly control feature informativeness or redundancy. Furthermore, these approaches often neglect the intrinsic topological structure encoded in assembly graphs, which captures valuable contig–contig relationships, crucial for accurate genome binning [30].

To address the aforementioned challenges, and inspired by recent advances in graph representation learning and canonical correlation analysis [31,32], we propose MBGCCA (Metagenomic Binning using Graph-based Canonical Correlation Analysis), a novel framework that integrates graph neural networks (GNNs) [33] with contrastive learning to improve contig binning performance. MBGCCA explicitly models the assembly graph to capture structural dependencies between contigs, and jointly optimizes feature embeddings using a contrastive objective that encourages proximity between structurally or functionally related contigs while increasing separation among unrelated ones in the latent space. By harmonizing graph topology with multimodal feature correlation, MBGCCA not only improves binning performance in terms of accuracy, robustness, and generalization, but also implicitly incorporates information-theoretic regularization through contrastive learning and CCA-based embedding decorrelation. This promotes high-entropy, low-redundancy representations that more effectively capture the underlying genomic structure.

## 2. Materials and Methods

### 2.1. Computational Framework of MBGCCA

**Figure 1:**
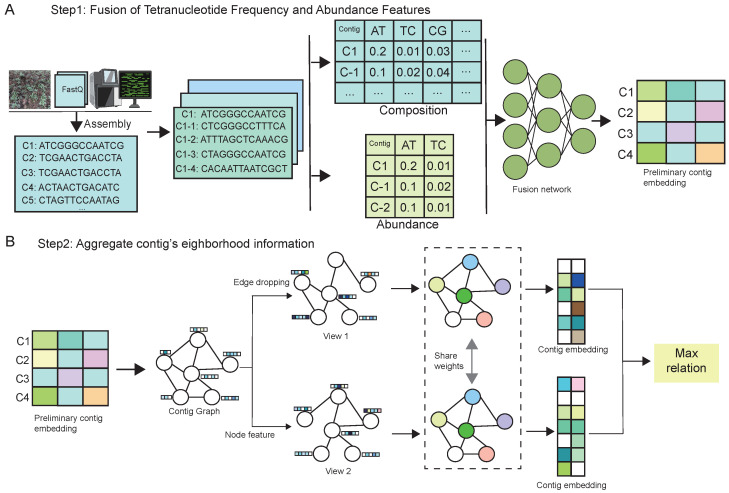
Overview of the MBGCCA framework. (**A**) Step 1: Fusion of TNF and abundance features. (**B**) Step 2: Aggregation of contig neighborhood information.

The computational framework of MBGCCA comprises two primary stages, as illustrated in Figure 1.

The MBGCCA framework operates in two sequential stages, each designed to address key challenges in metagenomic binning by integrating compositional, abundance, and structural information.

Stage one focuses on the fusion of two fundamental features: tetranucleotide frequency (TNF) and abundance profiles. Initially, assembled genome contigs are length-normalized through extension to ensure consistency in input size. TNF features capture sequence compositional patterns, while abundance profiles reflect sample-specific sequencing depth. These features are processed through a dedicated feature fusion network that generates high-dimensional embeddings, effectively encoding both structural and quantitative signals. This multimodal representation enhances the model’s ability to distinguish contigs from different microbial genomes based on both their intrinsic sequence characteristics and their relative abundance.

Stage two incorporates neighborhood context through a GNN module, which models the topological structure among contigs. To prevent overfitting and improve generalization, stochastic edge dropout is applied during training. Two perturbed graph views are generated and independently updated via message-passing mechanisms, allowing for local aggregation of neighborhood information. The resulting node embeddings are then aligned using CCA, which encourages maximum correlation between the two views. This contrastive alignment strategy facilitates the clustering of contigs from the same genome while promoting separation from unrelated contigs in the latent space.

By jointly optimizing multimodal feature integration and structure-aware representation learning, MBGCCA significantly improves binning performance. The final genome bins demonstrate enhanced biological coherence, benefiting from both topology-aware modeling and entropy-aware representation learning, which together enable robust and generalizable binning across diverse and complex microbial datasets. The overall framework jointly leverages contrastive learning and mutual information regularization in two stages: feature fusion and structural embedding. These learning objectives improve both the intra-contig consistency and inter-contig separation required for accurate genome binning.

### 2.2. Fusion of Tetranucleotide Frequency and Abundance Features

The initial step of the MBGCCA framework, illustrated in Figure 1A, focuses on multimodal feature integration through a feature fusion module. The overall process consists of four main components.

#### 2.2.1. Data Augmentation

To enhance the robustness and generalizability of the model, we apply a Random Subsequence Extraction strategy. For each contig in the dataset, five additional augmented versions are generated by randomly cropping subsequences of varying lengths. This simulates the incomplete or fragmented nature of contigs that commonly arise in real-world metagenomic assemblies due to sequencing errors or low coverage. As a result, each original contig is expanded into six views: the original sequence and five augmented variants. These multiple views are later used to reinforce the model’s ability to generalize across contig length variability and handle short or noisy sequences.

#### 2.2.2. TNF Calculation and Normalization

Following the methodology outlined by Teeling et al. [34] and Wang et al. [35], tetranucleotide frequencies (TNF) are calculated to construct compositional feature vectors for DNA contigs, providing a sequence-based signal that is complementary to coverage-based abundance features.

Step 1: K-mer Extraction. For each contig, a sliding window of size k=4 with stride 1 bp is applied to generate all possible overlapping tetranucleotides. For a contig of length $L_{i}$, this produces $\left(L_{i}-k+1\right)$ 4-mers. For example, the sequence “ATGCATG” yields the 4-mers “ATGC”, “TGCA”, “GCAT”, and “CATG”.

Step 2: Reverse Complement (RC) Merging. Because DNA is double-stranded, a 4-mer and its reverse complement carry identical biological information. To eliminate strand bias, each 4-mer is merged with its reverse complement into a canonical representation. For instance, “ATGC” and “GCAT” are counted as a single canonical k-mer. Palindromic k-mers are self-complementary and thus counted once. This reduces the dimensionality from 256 raw 4-mers to 136 canonical 4-mers.

Step 3: Frequency Computation. Let $N_{i,j}$ denote the count of the *j*-th canonical 4-mer in the *i*-th contig. The raw relative frequency is computed as

(1) $$f_{i,j}=\frac{N_{i,j}}{L_{i}-k+1},$$

which normalizes by the total number of 4-mers in the contig, thereby correcting for length-dependent bias.

Step 4: Smoothing. To reduce sparsity and avoid zero-valued features (especially in shorter contigs or rare k-mers), Laplace smoothing [36] is applied:

(2) $$f_{i,j}^{\prime }=f_{i,j}+1.$$

Step 5: Normalization. The smoothed frequencies are further normalized to form a probability distribution:

(3) $$q_{i,j}=\frac{f_{i,j}^{\prime }}{\sum_{k=1}^{136}\;f_{i,k}^{\prime }},$$

ensuring that feature vectors are comparable across contigs, regardless of length or coverage.

Step 6: Vector Construction. The final TNF feature vector for contig *i* is defined as

(4) $$x_{i}^{\left(\mathrm{com}\right)}=\;\left[q_{i,1},\;q_{i,2},\;\ldots ,\;q_{i,136}\right],$$

yielding a 136-dimensional representation of its compositional profile that is highly informative for downstream tasks such as genome binning, clustering, and embedding-based representation learning.

#### 2.2.3. Calculation of Abundance Features

The abundance feature of a contig is a key indicator reflecting the relative occurrence of genomic fragments in sequencing samples. Unlike compositional features that are derived from intrinsic nucleotide patterns, abundance features are estimated from sequencing depth profiles obtained by mapping reads back to assembled contigs. This provides a complementary source of information, as contigs belonging to the same genome typically exhibit similar coverage patterns across samples.

Step 1: Read Mapping and Depth Profiling. For each sample, raw sequencing reads are aligned to the assembled contigs using a short-read aligner such as Bowtie2 or BWA. This produces alignment files (SAM/BAM) that record how many reads map to each base position of each contig. The per-base coverage profile is then aggregated to compute mean coverage and variability. Specifically, the mean coverage quantifies the overall abundance of a contig in one sample, while the standard deviation reflects unevenness of coverage caused by sequencing bias or repeat regions.

Step 2: Coverage Vector Construction. For contig *i* across *M* samples, two parallel descriptors are extracted: the mean coverage vector

(5) $$C_{i}^{\left(\mathrm{mean}\right)}=\left(C_{i,1}^{\left(\mathrm{mean}\right)},C_{i,2}^{\left(\mathrm{mean}\right)},\ldots ,C_{i,M}^{\left(\mathrm{mean}\right)}\right),$$

and the standard deviation coverage vector

(6) $$C_{i}^{\left(\mathrm{std}\right)}=\left(C_{i,1}^{\left(\mathrm{std}\right)},C_{i,2}^{\left(\mathrm{std}\right)},\ldots ,C_{i,M}^{\left(\mathrm{std}\right)}\right),$$

where $C_{i,m}^{\left(\mathrm{mean}\right)}$ and $C_{i,m}^{\left(\mathrm{std}\right)}$ denote the average and variability of coverage of contig *i* in the *m*-th sample, respectively. Concatenating these two descriptors yields the raw abundance feature vector:

(7) $$C_{i}=\left(C_{i}^{\left(\mathrm{mean}\right)},C_{i}^{\left(\mathrm{std}\right)}\right).$$

This representation explicitly encodes both abundance levels and their stability across samples.

Step 3: Handling Missing Coverage. Some contigs may not be detected in certain samples, leading to zero coverage. To avoid issues caused by sparsity and division by zero, a small constant ϵ is added:

(8) $$C_{i,m}^{\prime }=C_{i,m}+\epsilon ,\;\epsilon =10^{-5}.$$

This smoothing step ensures numerical stability while preserving relative abundance differences.

Step 4: Cross-Sample Normalization. Because sequencing depth differs across samples, direct comparison of raw coverage values can be misleading. To ensure comparability, normalization is applied across all contigs within each sample:

(9) $$C_{i,m}^{\left(\mathrm{cov}\right)}=\frac{C_{i,m}^{\prime }}{\max_{k=1}^{N}C_{k,m}^{\prime }},$$

where *N* is the total number of contigs. This max-normalization rescales coverage values into [0,1], making contigs comparable across samples with different sequencing depths.

Step 5: Final Feature Representation. After smoothing and normalization, the final abundance feature vector for contig *i* is defined as

(10) $$x_{i}^{\left(\mathrm{abund}\right)}=\left(x_{i,1}^{\left(\mathrm{abund}\right)},x_{i,2}^{\left(\mathrm{abund}\right)},\;\ldots ,x_{i,2M}^{\left(\mathrm{abund}\right)}\right),$$

which is 2M-dimensional, combining both mean and variability descriptors for each of the *M* samples. This enriched representation captures not only the overall abundance profile of each contig but also its heterogeneity across sequencing datasets. When integrated with compositional TNF features, the abundance vector provides a robust foundation for downstream machine learning tasks such as clustering, genome binning, and multimodal representation learning.

#### 2.2.4. Fusion Network Construction

The objective function guides the optimization of the MBGCCA neural network, with the core being the Normalized Temperature-scaled Cross-Entropy [37] (NT-Xent) loss function. This contrastive learning-based loss function optimizes instance representations by pulling similar instances closer and pushing dissimilar ones apart.

During training, different views of the same contig are treated as positive samples, while views from other contigs are treated as negative samples. Specifically, suppose that each contig has *V* different views in a batch of size $N_{\mathrm{bs}}$, then the NT-Xent loss is defined as

(11) $$\begin{array}{c} L_{\mathrm{NT}-\mathrm{Xent}}=-\frac{1}{N_{\mathrm{bs}}V\left(V-1\right)}\sum_{i=1}^{N_{\mathrm{bs}}}\sum_{v=1}^{V}\sum_{\begin{array}{c} v_{1}=1\\ v_{1}\neq v \end{array}}^{V}\log\frac{\exp\left(\frac{\cos(z_{i,v},z_{i,v_{1}})}{\tau }\right)}{\sum_{j=1}^{N_{\mathrm{bs}}}\sum_{v_{2}=1}^{V}\exp\left(\frac{\cos(z_{i,v},z_{j,v_{2}})}{\tau }\right)} \end{array}$$

Here, $z_{i,v}$ denotes the representation of the *i*-th contig under the *v*-th view, τ is the temperature parameter that controls the sensitivity of the contrastive learning, and cos(a,b) denotes the cosine similarity between two representations. The numerator captures the similarity between positive pairs (different views of the same contig), while the denominator aggregates similarities across all samples to form the contrastive objective.

Compared with traditional single-view contrastive learning, this multi-view approach enhances the robustness of binning by ensuring that different fragments of the same contig are correctly clustered together. It also avoids the information loss caused by relying on a single hand-crafted view. This objective enables MBGCCA to achieve accurate and stable contig classification in metagenomic binning. Moreover, the NT-Xent loss encourages high mutual information between positive pairs while implicitly reducing feature redundancy across views, serving as a form of information-theoretic regularization.

#### 2.2.5. Training Details of Step 1

During training, TNF vectors and abundance feature vectors are used as inputs. A fusion network is employed for feature learning, consisting of three fully connected layers with 2048, 2048, and 128 units, designed to integrate multi-modal feature information and improve contig separability. The network is implemented in PyTorch v1.9.1 and trained over 200 epochs with a batch size of 1024. To prevent overfitting, early stopping is adopted, dynamically halting training based on validation loss and enhancing model generalization. The optimization target is the NT-Xent loss (Equation (11)), which improves binning performance by maximizing similarity between different views of the same contig (positive pairs) while minimizing similarity with other contigs (negative pairs).

### 2.3. Aggregation of Contig Neighborhood Information

The assembly graph, typically represented in GFA (Graphical Fragment Assembly) format, serves as the core structural input to the graph neural network (GNN) model in metagenomic binning tasks. This graph is generated during the de novo genome assembly process, where sequencing reads—especially from long-read or hybrid platforms—are first aligned and then progressively assembled into contiguous sequences (contigs). Rather than producing a linear genome sequence, modern assemblers (e.g., metaFlye [38], SPAdes [39]) construct an assembly graph to capture the inherent ambiguities and structural complexity present in metagenomic datasets, such as genomic repeats, strain-level variation, and incomplete coverage.

During graph initialization, each contig is treated as a node, and the edges denote observed connections (e.g., overlaps or scaffold links) between contigs $c_{i}$ and $c_{j}$. The adjacency matrix $A\in R^{N\times N}$, where *N* is the number of contigs, is constructed accordingly. Each entry $A_{ij}$ reflects the edge weight between $c_{i}$ and $c_{j}$, determined by the read coverage supporting their connection. If no direct linkage exists, $A_{ij}=0$.

To mitigate the influence of sequencing depth variability and improve comparability across datasets, the edge weights in A are normalized. This normalization strategy ensures that connections supported by higher-confidence sequencing data exert proportionally greater influence during GNN training, while still controlling for sample-specific depth biases.

To further enhance the discriminative power of the GNN, MBGCCA adopts a GraphSAGE-based neighborhood sampling and aggregation strategy [40]. In this approach, each contig (node) updates its representation by sampling a fixed-size subset of its neighbors, rather than aggregating over all connected nodes. This makes the model computationally efficient and scalable to large assembly graphs. For each sampled neighborhood, MBGCCA applies mean aggregation to combine the features of neighboring contigs and integrates them with the central contig’s own features, enabling multi-hop message passing and capturing broader structural context.

Through message passing on the constructed graph, the GraphSAGE mechanism enables each contig node to iteratively update its representation by aggregating information from its local neighborhood. This process integrates sequence composition (e.g., TNF), abundance features, and topological context, resulting in robust and biologically coherent embeddings. Consequently, MBGCCA enhances binning accuracy, particularly in complex and heterogeneous metagenomic environments.

#### 2.3.1. Self-Supervised Graph Representation Learning with CCA

In recent years, GNNs have demonstrated significant success in modeling graph-structured data, particularly in the domain of self-supervised learning [41]. While traditional contrastive learning frameworks operate at the instance level to distinguish between different samples, GNN-based methods can adopt a non-contrastive strategy, such as CCA, to efficiently learn meaningful graph representations without the need for negative sampling.

The essence of self-supervised learning lies in the generation of diverse views of the data, enabling the model to learn view-invariant and semantically consistent representations. In the context of graph data, we adopt two widely used augmentation strategies to construct such views:

- Edge Dropping [42]: A subset of edges is randomly removed from the graph to simulate structural variations. This perturbation enhances the model’s ability to generalize to different graph topologies and encourages reliance on robust neighborhood structures.
- Node Feature Masking [43]: Portions of node features are randomly masked, introducing uncertainty in node attributes and forcing the model to avoid over-dependence on specific dimensions. This strategy improves robustness and promotes the learning of more generalizable representations.

These augmented views are subsequently encoded via shared or parallel GNN encoders, and their representations are aligned using CCA-based objectives [44] to maximize inter-view correlation. This approach enables the model to capture structural and semantic consistency across perturbations, thereby yielding high-quality graph embeddings suitable for downstream tasks such as clustering or classification.

At each training iteration, two augmentations, $t_{A}$ and $t_{B}$, are sampled from the transformation set T to generate two graph views:

(12) $$G_{A}=\left(X_{A},\;A_{A}\right)\;\mathrm{and}\;G_{B}=\left(X_{B},\;A_{B}\right)$$

A shared-parameter GNN encoder is used to extract node embeddings. The core principle of the GNN is to update node representations using the adjacency structure (Figure 1B), defined as

(13) $$Z=f_{\theta }\left(X,\;A\right)$$

where $X\in R^{N\times F}$ is the node feature matrix, *N* is the number of nodes, *F* is the feature dimension, $A\in R^{N\times N}$ is the adjacency matrix, $f_{\theta }$ is the GNN encoder with parameters θ, and $Z\in R^{N\times D}$ is the output embedding matrix with dimension *D*.

A two-layer Graph Convolutional Network (GCN) [45] is adopted as the encoder. The node representations are updated according to the following propagation rule:

(14) $$H^{\left(l+1\right)}=\sigma \left(\hat{A}H^{\left(l\right)}W^{\left(l\right)}\right)$$

where $H^{\left(l\right)}$ is the feature matrix at layer *l* with $H^{\left(0\right)}=X$, $\hat{A}=D^{-1/2}AD^{-1/2}$ is the symmetric normalized adjacency matrix, $W^{\left(l\right)}$ is the trainable weight matrix at layer *l*, and σ denotes the activation function (ReLU).

The final representations from the two augmented views are computed as

(15) $$Z_{A}=f_{\theta }\left(X_{A},\;A_{A}\right),\;Z_{B}=f_{\theta }\left(X_{B},\;A_{B}\right)$$

To prevent numerical instability, node embeddings are standardized:

(16) $$\tilde{Z}=\frac{Z-\mu (Z)}{\sigma (Z)}\times \sqrt{N}$$

where μ(Z) and σ(Z) are the mean and standard deviation of the embedding matrix *Z*, ensuring zero mean and $1/\sqrt{N}$ standard deviation across each dimension.

##### Objective Function

The graph neural network employed in this work adopts CCA as its optimization objective. The loss function [44] is defined as

(17) $$L_{\mathrm{CCA}}={∥{\tilde{Z}}_{A}-{\tilde{Z}}_{B}∥}_{F}^{2}+\lambda \left({∥{\tilde{Z}}_{A}^{⊤}{\tilde{Z}}_{A}-I∥}_{F}^{2}+{∥{\tilde{Z}}_{B}^{⊤}{\tilde{Z}}_{B}-I∥}_{F}^{2}\right)$$

The first term, ${∥{\tilde{Z}}_{A}-{\tilde{Z}}_{B}∥}_{F}^{2}$, is the invariance loss [46], which minimizes the difference between embeddings from two augmented views, encouraging the model to learn view-invariant features.

The second term, ${∥{\tilde{Z}}_{A}^{⊤}{\tilde{Z}}_{A}-I∥}_{F}^{2}+{∥{\tilde{Z}}_{B}^{⊤}{\tilde{Z}}_{B}-I∥}_{F}^{2}$, is the decorrelation loss [44], designed to reduce redundancy between different feature dimensions and ensure that each dimension captures distinct information. This decorrelation mechanism is inspired by information-theoretic principles, aiming to maximize the entropy of learned representations by enforcing low mutual information among embedding dimensions. The hyperparameter λ controls the strength of the decorrelation term. Empirical results show that setting λ between 0.0005 and 0.001 yields optimal performance.

#### 2.3.2. Training Details of Step 2

We set the training epochs to 100 for the self-supervised stage. The learning rates for the encoder and the linear classifier were set to 1 × 10^−3^ and 1 × 10^−2^, respectively. Weight decay was set to 0 for the encoder and 1 × 10^−4^ for the linear evaluator. The hidden and output dimensions of the encoder were both set to 512, and we used a two-layer GNN architecture. For graph augmentations, the drop edge ratio and drop feature ratio were both set to 0.2. All experiments were conducted using a single GPU unless otherwise specified. Upon convergence, the learned node embeddings capture robust structural and semantic patterns, making them suitable for a variety of downstream graph-based tasks, such as node classification, link prediction, and graph clustering.

### 2.4. Evaluation Metrics

In this study, bins (i.e., genome classification units) generated by GraphMB and other binning tools are evaluated using CheckM [47] (parameters: –reduced-tree, version 1.1.2) to assess both completeness and contamination. To eliminate redundancy, dereplication is performed using dRep [48]. During filtering, bins with completeness greater than 90% and contamination less than 5% are classified as high-quality (HQ) bins.

Additionally, for simulated datasets, AMBER [49] is employed to provide gold-standard evaluation by comparing predicted bins against reference labels. In this setting, several key metrics are used.

#### 2.4.1. Average Purity (AP)

Purity measures the proportion of contig length in a bin that originates from a single reference genome. The average purity across all bins is defined as

(18) $$\mathrm{AP}=\frac{1}{B}\sum_{i=1}^{B}\frac{\max_{j}\left(L_{i,j}\right)}{\sum_{j}L_{i,j}},$$

where *B* is the number of predicted bins, $L_{i,j}$ is the total length of contigs in bin *i* that map to reference genome *j*, and $\max_{j}\left(L_{i,j}\right)$ corresponds to the dominant genome assignment for that bin. A higher AP indicates lower contamination.

#### 2.4.2. Average Completeness (AC)

Completeness reflects the proportion of a reference genome recovered in binning. The average completeness is defined as

(19) $$\mathrm{AC}=\frac{1}{R}\sum_{j=1}^{R}\frac{\sum_{i}L_{i,j}}{G_{j}},$$

where *R* is the total number of reference genomes, $\sum_{i}L_{i,j}$ is the sum of contig lengths assigned to genome *j* across bins, and $G_{j}$ is the total genome length. A higher AC indicates that bins better capture the full genome content.

#### 2.4.3. Contamination

In the context of AMBER, contamination is related to purity and defined as

(20) Contamination=1−Purity.

#### 2.4.4. F1 Score

To capture a balance between purity and completeness, the F1 score is calculated for each bin as

(21) $$F1_{i}=\frac{2\cdot {\mathrm{Purity}}_{i}\cdot {\mathrm{Completeness}}_{i}}{{\mathrm{Purity}}_{i}+{\mathrm{Completeness}}_{i}},$$

and the overall score is averaged across all bins. This metric emphasizes bins that achieve both high purity and high completeness simultaneously.

#### 2.4.5. High-Quality (HQ) Bins

Following the MIMAG standard, bins are defined as HQ if they satisfy

Completeness≥90%,Contamination≤5%.

The number of HQ bins is reported as a practical summary of binning performance.

#### 2.4.6. Dereplication and Uniqueness

During dereplication, dRep clusters highly similar bins (based on average nucleotide identity, ANI) into bin clusters. We define a bin as a unique HQ bin if it does not share a cluster with other HQ bins. This avoids redundancy and ensures the fair evaluation of distinct genome recovery.

#### 2.4.7. Consensus Integration

Finally, to integrate the strengths of different binning tools, DASTool [50] is used to combine bins from all methods into a consolidated set, which improves both the quality and reliability of the final binning results.

### 2.5. Datasets

#### 2.5.1. Pretraining Datasets

In this study, we aim to pretrain our model to improve the representation capacity of contigs, reduce reliance on manual feature engineering, and enhance binning performance for low-coverage and short-fragment contigs. Pretraining also enables the model to better capture underlying genomic patterns, thereby increasing its adaptability to diverse metagenomic datasets, especially in the context of uncultured microbial binning, ultimately boosting generalization, robustness, and efficiency.

We selected six datasets for training, including four simulated datasets and two real-world datasets. The four simulated datasets were provided by the CAMI II [51] challenge organizers (https://cami-challenge.org) (accessed on 20 August 2025), covering diverse microbial ecosystems: CAMI Mouse Gut (mouse gut microbiome), CAMI Skin (human skin microbiome), CAMI Airways (human airway microbiome), CAMI Gastrointestinal Tract (abbreviated as CAMI Gt).

To ensure accuracy in binning, gold standard cross-sample assemblies were used. The two real datasets include STEC, a collection of 53 fecal samples from the European Nucleotide Archive (ENA) project (https://www.ebi.ac.uk/ena/browser/view/PRJEB1775) (accessed on 10 April 2013) and Water Group1, a river water microbiome dataset with 8 samples, (retrieved from https://www.ebi.ac.uk/ena/browser/view/PRJNA542960) (accessed on 5 October 2019), mainly used to test performance under low-sample-count conditions.

#### 2.5.2. Benchmark Datasets

To comprehensively evaluate the performance of MBGCCA in metagenomic binning tasks, we conducted experiments on a diverse set of datasets:

- Simulated dataset: The simulated dataset Strong100 was generated using the Badread tool [52], following the methodology proposed by Quince et al. [53]. Specifically, sequencing reads were simulated from 100 bacterial strains representing 50 different species, with abundances assigned randomly to mimic natural community variability.
- Real wastewater treatment plant (WWTP) datasets: Hjor, Viby, Damh, Mari, AalE, and Hade.
- Real environmental dataset: Soil, representing complex microbial communities in natural soil environments.

To benchmark MBGCCA on long-read metagenomic binning, we generated synthetic long-read datasets using badread [52], following the simulation protocol adopted in LRBinner [54]. Specifically, reads were simulated from the 100 strains of Strong100 with randomly assigned abundances, and the assemblies were produced using metaFlye v2.9 [38] to ensure consistency and comparability with baseline methods.

Details of all test datasets are summarized in Table 1.

WWTP datasets were derived from Singleton et al. [55] (PRJNA629478). For each WWTP dataset, contig coverage was calculated from long-read assemblies, and three additional short-read samples from the same site (different time points) were used to compute contig abundance. Assemblies were generated using metaFlye, polished in three rounds with Racon v1.3.3 [56], and further corrected using short reads. Only one sample per WWTP dataset contains long-read data (used for assembly), while the other three are used for abundance estimation. The Soil dataset comes from Brunbjerg et al. [57], and was used alongside other datasets for model development and hyperparameter optimization—except for Damh and Hade, which were held out to evaluate potential overfitting.

##### Long-Read Simulation Protocol

For the simulation of long-read metagenomic data, we sampled strain-level abundances from a Dirichlet distribution, where $p_{g}$ denotes the proportion of reads assigned to strain *g*, and the total number of simulated bases was set to N=7.5 Gbp. The synthetic sequencing reads were generated using badread [52] with the following configuration:

- Mean read length: 10,000 bp.
- Standard deviation: 7000 bp.
- Read identity: mean = 98%; max = 99.9%; standard deviation = 5.
- Error model: nanopore2020 (default).

To emulate real-world long-read metagenomic workflows, all reads were assembled using metaFlye v2.9 [38], thereby ensuring high simulation fidelity and comparability across methods.

## 3. Results

In this study, we compared the performance of MBGCCA with five mainstream binning tools on the same datasets, including GraphBin [58], MaxBin2 [19], SemiBin-ocean [59], SemiBin-train [59], VAMB [27], MetaBAT2 [21], and GraphMB [28], using default parameters for all methods. All methods took contig sequences and their abundance information as input.

### 3.1. Evaluation on Simulated Dataset Strong100

We evaluated MBGCCA and baseline methods on the simulated dataset Strong100 using the AMBER toolkit [49]. Evaluation metrics included average purity (AP, bp-weighted), average completeness (AC, bp-weighted), F1 score (harmonic mean of AP and AC), and the number of high-quality (HQ) bins (completeness > 90%, contamination < 5%).

As shown in Table 2, MBGCCA consistently outperforms all competing methods across key metrics. It achieves the highest AP of 0.986, outperforming VAMB (0.969) and GraphMB (0.967), indicating superior ability in reducing contamination and improving bin purity. For AC, MBGCCA attains 0.784, slightly surpassing SemiBin-ocean (0.783) and significantly exceeding GraphBin (0.613) and MetaBAT2 (0.592), demonstrating robust genome recovery. MBGCCA also achieves the highest F1 score (0.864), outperforming GraphMB (0.852) and VAMB (0.849), and recovers the most HQ bins (32), a 10.3% improvement over the next-best method (GraphMB, 29).

While MBGCCA adopts a self-supervised learning strategy without requiring labeled genomes, methods like SemiBin-train rely on pretraining with external annotations. Despite this, MBGCCA still surpasses SemiBin-train across all metrics (AP: 0.986 vs. 0.826; F1: 0.864 vs. 0.823; HQ bins: 32 vs. 20). This highlights MBGCCA’s strength in generalizability and scalability, particularly in environments where reference genomes are unavailable or incomplete. We have emphasized this contrast in the Section 4.

In summary, MBGCCA achieves top performance across all four core metrics—AP, AC, F1, and HQ bins—solidifying its effectiveness and reliability for microbial genome reconstruction. Its consistent accuracy makes it well-suited for metagenomic analysis in complex or low-abundance microbial environments.

### 3.2. Evaluation on Real-World Datasets

Table 3 presents the number of high-quality (HQ) bins produced by various binning tools across multiple real-world datasets, including GraphBin, MaxBin2, SemiBin-ocean, SemiBin-train, VAMB, MetaBAT2, GraphMB, and the proposed method, MBGCCA. The number of HQ bins serves as a key indicator for assessing the effectiveness of each binning method on different datasets.

As shown in Table 3, MBGCCA achieves the highest number of HQ bins across all datasets, outperforming all other tools. For example, in the Hjor, Viby, Damh, and Hade datasets, MBGCCA obtains 28, 32, 46, and 56 HQ bins respectively, exceeding all competitors.

In the Hade dataset, MBGCCA achieves 56 HQ bins, representing a 27.3% increase over MetaBAT2 (44 bins) and a 7.7% improvement over GraphMB (52 bins), highlighting its superior performance in HQ bin identification. In the AalE dataset, MBGCCA and MetaBAT2 both achieve 43 HQ bins, substantially outperforming VAMB and GraphMB, indicating MBGCCA’s robustness and consistency across datasets. Although MetaBAT2 is widely regarded as a strong binning tool, MBGCCA consistently matches or exceeds its performance in all scenarios. For instance, on the Viby dataset, MBGCCA yields 32 HQ bins compared to MetaBAT2’s 29, an improvement of 10.3%. On the Damh dataset, MBGCCA produces 46 HQ bins versus MetaBAT2’s 41, a 12.2% increase.

These results demonstrate that MBGCCA offers superior or at least equivalent performance compared to mainstream binning tools, with generally higher HQ bin counts, supporting its effectiveness, accuracy, and robustness in microbial genome reconstruction across diverse real-world environments.

### 3.3. Runtime and Memory Usage Analysis of MBGCCA

We evaluated the runtime and peak memory usage of three deep learning-based binning tools on the Soil dataset. All tools were executed on a workstation equipped with dual AMD EPYC 7H12 64-Core Processors and an NVIDIA RTX 4090 GPU. Each tool was run using 48 threads, and the time spent on aligning reads to contigs was excluded, as this step is required by all binning methods.

Each method was executed three times per dataset, and the average runtime and memory usage were recorded.

As illustrated in Table 4 and Table 5, MBGCCA exhibits moderate increases in runtime and memory usage compared to other deep learning-based binning methods. Specifically, MBGCCA’s runtime is slightly longer than VAMB but shorter than GraphMB on both Hade and Soil datasets. In terms of peak memory usage, MBGCCA consumes more memory than both VAMB and GraphMB, especially on the larger Soil dataset (10.5 GB).

Despite these resource requirements, MBGCCA achieves significantly higher binning performance (Table 1 and Table 2), particularly in terms of genome completeness, purity, and recovery of low-abundance genomes. These gains stem from the integration of multi-modal features, graph-based neighborhood aggregation, and contrastive learning strategies. Thus, the slightly increased computational cost is a worthwhile trade-off for its enhanced accuracy and robustness in complex metagenomic scenarios.

### 3.4. Ablation Study

To evaluate the contribution of different input features and model components in the MBGCCA framework, we conducted an ablation study with the following variants. GraphMB (TNF): This variant uses only tetranucleotide frequency (TNF) features as input, excluding abundance information, to assess the compositional contribution. GraphMB (Abundance): This model uses only abundance profiles without TNF features to evaluate the impact of sequencing-depth-based information. GraphMB (Step 1): This version retains the multimodal feature fusion module and uses the initial contig embeddings from stage one, but excludes neighborhood aggregation via the graph neural network. As such, it does not incorporate contig–contig topological relationships and serves to isolate the effectiveness of the graph-based learning in stage two. These variants allow us to dissect the individual impact of input features and structural information, and to better understand the importance of multimodal integration and graph representation learning in our model.

The results (Table 6) demonstrate that while both compositional (TNF) and abundance-based features contribute to performance, their combination significantly improves binning outcomes. Notably, GraphMB (Step 1) outperforms single-feature variants, confirming the utility of multimodal feature fusion. The full MBGCCA model achieves the highest number of HQ bins across all datasets, highlighting the critical role of graph-based neighborhood aggregation in enhancing genome recovery, especially in complex microbial environments such as soil.

## 4. Discussion

In this study, we proposed a metagenomic binning method based on assembly graphs, termed MBGCCA, and conducted comprehensive evaluations on both simulated and real-world environmental datasets. Compared with conventional binning approaches, MBGCCA integrates GNNs with contrastive learning and information-theoretic regularization, enabling a more principled incorporation of assembly graph topology and genomic features. Experimental results demonstrate that MBGCCA not only performs well on simulated assemblies, but also exhibits strong generalization and adaptability to complex environmental metagenomic data.

Current metagenomic binning methods primarily rely on the fusion of sequence composition features (such as TNF) and abundance coverage. However, these approaches face challenges in recovering low-abundance species, handling noise in complex environments, and adapting to long-read sequencing data. MBGCCA addresses these limitations by leveraging GNNs to model the assembly graph and applying contrastive learning with entropy-aware objectives to optimize the embedding space. This allows the model to better capture topological structure while maximizing the information content of learned representations. The assembly graph encodes connectivity between contigs, and MBGCCA utilizes these connections to ensure that contigs belonging to the same genome are accurately grouped.

Traditional methods based on TNF and abundance are often sensitive to environmental noise and produce redundant or low-informative embeddings. In contrast, MBGCCA improves robustness by introducing decorrelation loss, which reduces feature redundancy and encourages the learning of high-entropy, information-rich representations. This entropy-aware design enhances both discriminative power and generalization ability across heterogeneous samples. Additionally, MBGCCA incorporates multi-view data augmentation and mutual information maximization to better recover low-abundance microbes, leading to improved performance on real-world metagenomic datasets.

Although MBGCCA offers improved computational efficiency over GraphMB, it still incurs higher computational costs on ultra-large datasets such as Soil. Future work may explore lightweight GNN architectures to reduce resource consumption. Currently, MBGCCA primarily utilizes genomic topology, TNF, and abundance features. Future extensions may incorporate multi-omics data, such as metatranscriptomics and metabolomics, to further enhance the information-theoretic richness and biological interpretability of the learned embeddings. The current study focuses on assembled contig sequences. Further optimization could extend MBGCCA to support novel data modalities, such as single-cell sequencing and spatial transcriptomics, thereby broadening its utility and resolution in microbial genome analysis.

## 5. Conclusions

In this study, we presented MBGCCA, a novel metagenomic binning framework that combines graph neural networks, contrastive learning, and information-theoretic regularization to improve the accuracy and robustness of genome reconstruction from complex metagenomic data. By leveraging assembly graph topology and integrating compositional and abundance features, MBGCCA generates high-entropy, low-redundancy embeddings that more faithfully represent the underlying genomic structure. Our experiments on both synthetic and real-world environmental datasets demonstrate that MBGCCA consistently outperforms existing binning methods, particularly in recovering low-abundance genomes and handling noisy, heterogeneous samples. The incorporation of entropy-aware objectives, such as mutual information maximization and embedding decorrelation, further enhances the discriminative power and generalization of the model. Future extensions of MBGCCA may explore lightweight GNNs, integration with multi-omics data, and support for new data modalities such as single-cell and spatial metagenomics, thereby expanding its applicability to a broader range of microbiome research scenarios.

## Figures and Tables

**Table 1:** Test datasets overview.

Dataset	Total	Reads N50	Assembly	Contigs N50	Mean	Contigs	Edges	Samples
	(Gbp)	(kbp)	(Gbp)	(kbp)	Cov.			
Strong100	7.5	13.3	0.17	175.0	42	852	670	1
Hjor	16.0	8.7	0.86	80.4	13	19,496	5937	4
Viby	17.2	14.0	1.32	101.0	7	23,389	7800	4
Damh	26.7	14.3	1.93	119.0	8	32,771	14,066	4
Mari	23.3	10.1	1.69	83.1	8	36,611	12,651	4
AalE	27.7	10.2	1.92	83.4	8	40,827	12,425	4
Hade	45.2	9.8	3.01	73.9	9	70,402	27,952	4
Soil	115.0	7.7	1.98	93.3	19	51,135	69,522	1

**Table 2:** AMBER assessment metrics for binning tools on the simulated Strong100 dataset.

Method	AP (bp)	AC (bp)	F1	HQ Bins
GraphBin	0.848	0.613	0.712	23
MaxBin2	0.818	0.765	0.791	14
SemiBin-ocean	0.858	0.783	0.819	26
SemiBin-train	0.826	0.820	0.823	20
VAMB	0.969	0.755	0.849	26
MetaBAT2	0.905	0.592	0.716	26
GraphMB	0.967	0.762	0.852	29
MBGCCA	0.986	0.784	0.864	32

Note: Higher values of AP (average purity), AC (average completeness), F1 score, and HQ bins (high-quality bins) indicate better performance.

**Table 3:** Comparison of HQ bins obtained using MBGCCA and state-of-the-art binning tools.

Method	Hjor	Viby	Damh	Mari	AalE	Hade	Soil
GraphBin	11	15	14	16	12	6	0
MaxBin2	12	19	16	14	12	19	0
SemiBin-ocean	11	1	22	18	21	7	0
SemiBin-train	7	4	23	22	32	25	0
VAMB	22	12	22	30	37	8	0
MetaBAT2	23	29	41	39	43	44	2
GraphMB	25	23	43	48	**46**	52	**3**
MBGCCA	**28**	**32**	**46**	**49**	43	**56**	**3**

**Table 4:** Runtime comparison (in seconds) of three deep learning-based methods.

Method	Hade (s)	Soil (s)
VAMB	1323	1241
GraphMB	2045	3768
MBGCCA	1832	3387

**Table 5:** Peak memory usage (in MB) of three deep learning-based methods.

Method	Hade (MB)	Soil (MB)
VAMB	1564	1322
GraphMB	1902	9343
MBGCCA	2067	10,532

**Table 6:** Comparison of HQ bins obtained using different variants of MBGCCA.

Method	Hjor	Viby	Damh	Mari	AalE	Hade	Soil
GraphMB (TNF)	20	23	18	16	22	28	1
GraphMB (Abundance)	14	18	19	17	19	16	0
GraphMB (Step 1)	24	26	40	42	40	48	2
MBGCCA	**28**	**32**	**46**	**49**	**43**	**56**	**3**

## Data Availability

The data presented in this study are openly available in the European Nucleotide Archive (ENA) under project accession PRJEB1775 (https://www.ebi.ac.uk/ena/browser/view/PRJEB1775).
